
JOURNAL INFORMATION
==============================
NLM Title Abbreviation: J High Energy Phys
Journal ID: J High Energy Phys
NLM Journal ID: 101668727

Journal of High Energy Physics

ISSN: 1126-6708
EISSN: 1029-8479

ARTICLE INFORMATION
==============================
PMCID: PMC8827129
PMID: 35226719
DOI: 10.1007/JHEP07(2016)009
Article ID: 4265
Article version: 1
Subjects: Erratum

Erratum to: Probing colored particles with photons, leptons, and jets

Kats Yevgeny kats@physics.rutgers.edu

Strassler Matthew J. strassler@physics.rutgers.edu

grid.430387.b 0000000419368796 New High Energy Theory Center, Department of Physics and Astronomy, Rutgers University, Piscataway, NJ 08854 U.S.A.
Electronic publication date: 2016 Jul 4
Print publication date: 2016
Volume: 2016
Issue: 7
Electronic Location ID: 9
Received 2016 Jun 25; Accepted 2016 Jun 27
Copyright: © The Author(s) 2016
License: Open AccessThis article is distributed under the terms of the Creative Commons Attribution 4.0 International License (https://creativecommons.org/licenses/by/4.0), which permits use, duplication, adaptation, distribution, and reproduction in any medium or format, as long as you give appropriate credit to the original author(s) and the source, provide a link to the Creative Commons license, and indicate if changes were made.
License URL: https://creativecommons.org/licenses/by/4.0/

Erratum for: 10.1007/JHEP11(2012)097
issue-copyright-statement: © SISSA, Trieste, Italy 2016

==============================
Open Access

This article is distributed under the terms of the Creative Commons Attribution License (CC-BY 4.0), which permits any use, distribution and reproduction in any medium, provided the original author(s) and source are credited.

Erratum to: JHEP11(2012)097

ArXiv ePrint: 1204.1119

